# A Quantum Walk Model for Idea Propagation in Social Network and Group Decision Making

**DOI:** 10.3390/e23050622

**Published:** 2021-05-16

**Authors:** Qizi Zhang, Jerome Busemeyer

**Affiliations:** 1 Department of Mechanical & Aerospace Engineering, Missouri University of Science and Technology, Rolla, MO 65409, USA; 2 Department of Psychological and Brain Sciences, Indiana University—Bloomington, Bloomington, IN 47405, USA; jbusemey@indiana.edu

**Keywords:** quantum walk, group decision making, idea propagation, social network

## Abstract

We propose a quantum walk model to investigate the propagation of ideas in a network and the formation of agreement in group decision making. In more detail, we consider two different graphs describing the connections of agents in the network: the line graph and the ring graph. Our main interest is to deduce the dynamics for such propagation, and to investigate the influence of compliance of the agents and graph structure on the decision time and the final decision. The methodology is based on the use of control-*U* gates in quantum computing. The original state of the network is used as controller and its mirrored state is used as target. The state of the quantum walk is the tensor product of the original state and the mirror state. In this way, the proposed quantum walk model is able to describe asymmetric influence between agents.

## 1. Introduction

The investigations of group decision making and social networks have regularly attracted contributions from applied mathematicians, psychologists, and social scientists over the last several decades. Graph theory and matrix algebra have natural applications to such investigations; for example, see the early monograph by Harary, Norman, and Cartwright [1]. Classic problems of interest include comparative static analyses of social network structures [2,3], functional implications of network structures [4], and numerical taxonomies of nodes [5,6]. Much ongoing interest is focusing on dynamic models of structural change [7,8,9,10,11] and on a broad range of dynamic processes unfolding over static networks; examples include the study of social learning [12,13], opinion formation [14,15], and information propagation [4,8,16]. The study of dynamic models directly addresses one of the key problems of the field, which is to understand the implications of social structures for relevant dynamical states of the network.

Many classical random walk models have been developed for human networks (see the survey of Masuda et al. [17]). Propagation of ideas in social network and discussions in group decision making involves human cognition. Numerous empirical findings in cognitive psychology and behavioral sciences have exhibited anomalies with respect to the classical benchmark defined by Kolmogorovian probability axioms and the rules of Boolean logic, suggesting a whole new scope of research: the development of models for decision-making based on the quantum formulation of probability theory [18,19,20,21,22]. In order to account for the quantum feature of human cognition and decision making, a quantum walk model is proposed in this work to model the formation of agreement in group decision making and the propagation of ideas in social network.

Various quantum walk models have been proposed for information propagation in network and decision making [23,24,25,26]. Cabello et al. [27 ] proposed a quantum model for social network. Their quantum model can model the agent connections with various properties (such as hobby, relationship, etc.). However, their quantum model cannot model the dynamic information exchange between agents. Mutlu and Garibay [28 ] proposed a quantum-like approach for the analysis of social contagion dynamics with heterogeneous adoption thresholds. Khrennikov [29] proposed a social laser model for the bandwagon effect. The variable investigated in their model is the fraction of individuals in the network adopting an idea. Therefore, their models cannot model the fine-grained interaction between two particular agents. Recently, Bagarello et al. propose a simple approach based on ladder fermionic operators to investigate the spreading of news (may be true or biased by agent) in a social network [30]. All the these previously proposed quantum walks have the limitation that they are unable to model asymmetric transitions. The reason for such a limitation is that all these quantum walks are directly based on the Schrodinger equation or Heisenberg equation. Since the Hamiltonian (for continuous time quantum walks) is Hermitian and the associated operator is unitary, the transitions modeled by these quantum walks must be symmetric, that is, idea propagation from agent 1 to agent 2 is as easy as that from agent 2 to agent 1. However, idea propagations in practical social networks are always asymmetric. For example, a stubborn person may easily persuade an open-minded person, but an open-minded person may not persuade a stubborn person. The quantum walk model proposed in this work overcomes the limitation of symmetry by lifting the state of the social network into a higher dimensional state (tensor product of the original state and its mirrored state) and applying controlled-operator on the lifted state, where the original state works as controller and the mirrored state works as target. In this way, local transition in the target can be asymmetric, although the global transition in the lifted state is still symmetric.

In this paper, we propose a lifted quantum walk model for formation of agreement in group decision making and idea propagation in the social network. In Section 2, we introduce the basics of quantum computing and develop the unitary operator driving the lifted quantum walk model using control-*U* gates. In Section 2.3, we give the initialization, iteration, and stopping rule of the quantum walk. In Section 3, we develop the lifted quantum walk specifically for a network with connections in a ring graph. In Section 4, we give numerical results for idea propagation in a line graph and formation of agreement and interference effect in group decision making. Finally, we present our concluding remarks and share some of our ideas for future work in Section 5.

## 2. Quantum Walk for Asymmetric Propagation

Suppose $A_{1}$, $A_{2}$, $A_{3}$ are agents and each agent may or may not be infected (by an idea) and one agent can spread the infection to another, but also an agent can remove an infection. Let *z* be $A_{3}$’s state, *y* be $A_{2}$’s state, and *x* be $A_{1}$’s state. Each agent has two states, namely, 0 (not infected) and 1 (infected). $A_{1}$ interacts with $A_{2}$ and $A_{2}$ interacts with $A_{3}$:

$$A_{1}⟷A_{2}⟷A_{3}$$

### 2.1. States of the Network and the Quantum Walk

The global state of the 3-agent network is denoted by zyx. Since each agent has two states, the network has eight states. Viewing zyx as an integer in binary number system, we can represent zyx with its corresponding decimal form plus 1:
12345678000001010011100101110111

We encode the local state of an agent with one qubit and encode the global state of the network with the tensor products of the qubits of all agents. Specifically, we encode 0 as |0〉 and encode 1 as |1〉. |0〉 and |1〉 can be represented by basis vectors in the Hilbert space $C^{2}$, where C is the set of all complex numbers:

(1) $$|0\rangle =\left[\begin{array}{c} 1\\ 0 \end{array}\right],|1\rangle =\left[\begin{array}{c} 0\\ 1 \end{array}\right].$$

Then |zyx〉=|z〉⊗|y〉⊗|x〉 is a basis vector of $C^{8}$. For example,

$$|6\rangle =|101\rangle =|1\rangle ⊗\left[\begin{array}{c} 1\times |1\rangle \\ 0\times |1\rangle \end{array}\right]=|1\rangle ⊗{\left[\begin{array}{cccc} 0 & 1 & 0 & 0 \end{array}\right]}^{T}={\left[\begin{array}{cccccccc} 0 & 0 & 0 & 0 & 0 & 1 & 0 & 0 \end{array}\right]}^{T}.$$

It would be clear from context whether |1〉 means [0 1]${}^{T}$ or |001〉. (Note the superscript T denotes transpose and not conjugate transpose).

We model each agent as a quantum subsystem and model the communication of ideas among the agents as interaction among the quantum subsystems. In quantum physics, the interaction among the subsystems is accounted in the Hamiltonian of the total system. In quantum computing, the interaction among qubits is implemented by exerting a quantum gate (represented by a unitary operator) on the joint state (tensor product of all qubits). Let the superposition state spanned by the basis |zyx〉 be |ψ〉. In order to account for the asymmetric interaction between the agents, the quantum walk is not run directly on the network, but rather, on the tensor product of the network and a mirror of itself, that is, the superposition state of the quantum walk is $\left|\psi \rangle ⊗\right|\psi^{\prime }\rangle$, which is represented as a 64×1 vector. The mirror system $|\psi^{\prime }\rangle$ is used as reference for the control gates. This is the so-called lifting operation [31,32], where each state is lifted to a higher-dimension state. In the case of idea propagation, we model idea as a state superpositioned over |1〉 (accepting the idea) and |0〉 (rejecting the idea). Although interactions between agents often involves information exchange, we do not explicitly model information as a state. Agent interactions are considered in the unitary operators and information exchange is also implicitly considered in the unitary operators.

### 2.2. Control-*U* Gate

The quantum model performs the asymmetric transitions by using a collection of control-*U* gates (If condition is present, then apply the *U* gate to the action). To form the control-*U* gate, we need the following two measurement operators for the local state of a single agent:

(2) $$M_{0}=\left[\begin{array}{cc} 1 & 0\\ 0 & 0 \end{array}\right],M_{1}=\left[\begin{array}{cc} 0 & 0\\ 0 & 1 \end{array}\right].$$

Throughout this work, $I_{n}$ is an n×n identity matrix.

Let $|\alpha_{C}\rangle =c_{0}\left|0\rangle +c_{1}\right|1\rangle$ be the control qubit and $|\alpha_{T}\rangle$ be the *n*-qubit target state. The control-*U* gate for the (n+1)-qubit state $|\alpha_{C}\rangle ⊗|\alpha_{T}\rangle$ is $M_{0}⊗I_{2^{n}}+M_{1}⊗U$, where *U* is a $2^{n}\times 2^{n}$ unitary matrix. This control-*U* gate simply transforms ${\left[\begin{array}{cc} c_{0}|\alpha_{T}\rangle & c_{1}|\alpha_{T}\rangle \end{array}\right]}^{T}$ into ${\left[\begin{array}{cc} c_{0}|\alpha_{T}\rangle & c_{1}U|\alpha_{T}\rangle \end{array}\right]}^{T}$, as is shown below.

(3) $$\left(M_{0}⊗I_{2^{n}}+M_{1}⊗U\right)\left(\right|\alpha_{C}\rangle ⊗\left|\alpha_{T}\rangle \right)=\left[\begin{array}{c} c_{0}|\alpha_{T}\rangle \\ 0_{n\times 1} \end{array}\right]+\left[\begin{array}{c} 0_{n\times 1}\\ c_{1}U|\alpha_{T}\rangle \end{array}\right]=\left[\begin{array}{c} c_{0}|\alpha_{T}\rangle \\ c_{1}U|\alpha_{T}\rangle , \end{array}\right]$$

where $0_{n\times 1}$ is a n×1 zero vector. When $|\alpha_{C}\rangle =|0\rangle$, that is, $c_{0}=1,c_{1}=0$, it happens that ${\left[\begin{array}{cc} c_{0}|\alpha_{T}\rangle & c_{1}|\alpha_{T}\rangle \end{array}\right]}^{T}$ = ${\left[\begin{array}{cc} c_{0}|\alpha_{T}\rangle & c_{1}U|\alpha_{T}\rangle \end{array}\right]}^{T}$. When $|\alpha_{C}\rangle =|1\rangle$, that is, $c_{0}=0,c_{1}=1$, the control-*U* gate simply transforms $\left|1\rangle ⊗\right|\alpha_{T}\rangle$ into $\left|1\rangle ⊗U\right|\alpha_{T}\rangle$. The *U* gate is turned off when $|\alpha_{C}\rangle =|0\rangle$ and is fully turned on when $|\alpha_{C}\rangle =|1\rangle$.

If we want the *U* gate to be turned off when $|\alpha_{C}\rangle =|1\rangle$ and fully turned on when $|\alpha_{C}\rangle =|0\rangle$, then the control-*U* gate for $|\alpha_{T}\rangle ⊗|\alpha_{C}\rangle$ should be $M_{0}⊗U+M_{1}⊗I_{2^{n}}$.

Similarly, the control-*U* gate for the (n+1)-qubit state $|\alpha_{T}\rangle ⊗|\alpha_{C}\rangle$ is $I_{2^{n}}⊗M_{0}+U⊗M_{1}$ where |0〉 turns *U* off and |1〉 turns *U* fully on. To see this, suppose $|\alpha_{T}\rangle ={\left[\begin{array}{cccc} t_{0} & t_{1} & \cdots & t_{2^{n}} \end{array}\right]}^{T}$ and $U|\alpha_{T}\rangle ={\left[\begin{array}{cccc} t_{0}^{\prime } & t_{1}^{\prime } & \cdots & t_{2^{n}}^{\prime } \end{array}\right]}^{T}$. Then

(4) $$\left(I_{2^{n}}⊗M_{0}+U⊗M_{1}\right)\left(\right|\alpha_{T}\rangle ⊗\left|\alpha_{C}\rangle \right)={\left[\begin{array}{ccccccc} c_{0}t_{0} & c_{1}t_{0}^{\prime } & c_{0}t_{1} & c_{1}t_{1}^{\prime } & \cdots & c_{0}t_{2^{n}} & c_{1}t_{2^{n}}^{\prime } \end{array}\right]}^{T}.$$

When $|\alpha_{C}\rangle =|0\rangle$, that is, $c_{0}=1,c_{1}=0$, it happens that $[\begin{array}{ccccc} c_{0}t_{0} & c_{1}t_{0}^{\prime } & c_{0}t_{1} & c_{1}t_{1}^{\prime } & \cdots \end{array}$$\begin{array}{cc} c_{0}t_{2^{n}} & c_{1}t_{2^{n}}^{\prime } \end{array}{]}^{T}={\left[\begin{array}{ccccccc} t_{0} & 0 & t_{1} & 0 & \cdots & t_{2^{n}} & 0 \end{array}\right]}^{T}$ = $|\alpha_{T}\rangle ⊗|0\rangle$. When $|\alpha_{C}\rangle =|1\rangle$, that is, $c_{0}=0,c_{1}=1$, it happens that ${\left[\begin{array}{ccccccc} c_{0}t_{0} & c_{1}t_{0}^{\prime } & c_{0}t_{1} & c_{1}t_{1}^{\prime } & \cdots & c_{0}t_{2^{n}} & c_{1}t_{2^{n}}^{\prime } \end{array}\right]}^{T}$ = [0 $t_{0}^{\prime }$ 0 $t_{1}^{\prime }$⋯ 0 $t_{2^{n}}^{\prime }$] = $U|\alpha_{T}\rangle ⊗|1\rangle$. The *U* gate is turned off when $|\alpha_{C}\rangle =|0\rangle$ and is fully turned on when $|\alpha_{C}\rangle =|1\rangle$. If we want the *U* gate to be turned off when $|\alpha_{C}\rangle =|1\rangle$ and fully turned on when $|\alpha_{C}\rangle =|0\rangle$, then the control-*U* gate for $|\alpha_{T}\rangle ⊗|\alpha_{C}\rangle$ should be $U⊗M_{0}+I_{2^{n}}⊗M_{1}$.

Let $|\alpha_{T1}\rangle$ be an *m*-qubit state and $|\alpha_{T2}\rangle$ be an *n*-qubit state. Let the $2^{m}\times 2^{m}$ unitary matrix $U_{1}$ be the control operator for $|\alpha_{T1}\rangle$ and the $2^{n}\times 2^{n}$ unitary matrix $U_{2}$ be the control operator for $|\alpha_{T2}\rangle$. The control-*U* gate for the (m+n+1)-qubit state $|\alpha_{T1}\rangle ⊗\left|\alpha_{C}\rangle ⊗\right|\alpha_{T2}\rangle$ is $I_{2^{m}}⊗M_{0}⊗I_{2^{n}}+U_{1}⊗M_{1}⊗U_{2}$ where |0〉 turns *U* off and |1〉 turns $U_{1}$ and $U_{2}$ fully on. To see this, suppose $|\alpha_{T1}\rangle ={\left[\begin{array}{cccc} t_{0} & t_{1} & \cdots & t_{2^{m}} \end{array}\right]}^{T}$ and $U_{1}|\alpha_{T1}\rangle ={\left[\begin{array}{cccc} t_{0}^{\prime } & t_{1}^{\prime } & \cdots & t_{2^{m}}^{\prime } \end{array}\right]}^{T}$. Then

(5) $$\begin{array}{cc} \; & \left(I_{2^{m}}⊗M_{0}⊗I_{2^{n}}+U_{1}⊗M_{1}⊗U_{2}\right)\left(\right|\alpha_{T1}\rangle ⊗|\alpha_{C}\rangle ⊗\left|\alpha_{T2}\rangle \right)\\ = & |\alpha_{T1}\rangle ⊗\left[\begin{array}{c} c_{0}\\ 0 \end{array}\right]⊗|\alpha_{T2}\rangle +\left(U_{1}|\alpha_{T1}\rangle \right)⊗\left[\begin{array}{c} 0\\ c_{1} \end{array}\right]⊗\left(U_{2}|\alpha_{T2}\rangle \right)\\ = & {\left[\begin{array}{ccccccc} c_{0}t_{0}|\alpha_{T2}\rangle & c_{1}t_{0}^{\prime }U_{2}|\alpha_{T2}\rangle & c_{0}t_{1}|\alpha_{T2}\rangle & c_{1}t_{1}^{\prime }U_{2}|\alpha_{T2}\rangle & \cdots & c_{0}t_{2^{n}}|\alpha_{T2}\rangle & c_{1}t_{2^{n}}^{\prime }U_{2}|\alpha_{T2}\rangle \end{array}\right]}^{T}. \end{array}$$

The unitary operator driving the quantum walk is proposed in the block diagonal form:

(6) $$U=\mathrm{diag}(\left[\begin{array}{cccc} U_{1} & U_{2} & \cdots & U_{8} \end{array}\right]),$$

where each $U_{i}$ is an 8×8 matrix. The basis of $\left|\psi \rangle ⊗\right|\psi^{\prime }\rangle$ is $\left|xyz\rangle ⊗\right|x^{\prime }y^{\prime }z^{\prime }\rangle$. We label |xyz〉 so that it ranges from |1〉 to |8〉 and label $|x^{\prime }y^{\prime }z^{\prime }\rangle$ so that it ranges from $|1^{\prime }\rangle$ to $|8^{\prime }\rangle$. Then $\left|xyz\rangle ⊗\right|x^{\prime }y^{\prime }z^{\prime }\rangle$ ranges from $\left|1\rangle ⊗\right|1^{\prime }\rangle$, $\left|1\rangle ⊗\right|2^{\prime }\rangle$, …, to $\left|8\rangle ⊗\right|8^{\prime }\rangle$, with the only nonzero entry 1 moving down the column. To see the intricate operation of *U* on $\left|\psi \rangle ⊗\right|\psi^{\prime }\rangle$, we write $\left|\psi \rangle ⊗\right|\psi^{\prime }\rangle$ also in a block form:

(7) $$\left|\psi \rangle ⊗\right|\psi^{\prime }\rangle =\sum_{i=1}^{8}c_{i}|i\rangle ⊗\sum_{j=1}^{8}d_{j}|j^{\prime }\rangle =\sum_{i=1}^{8}\sum_{j=1}^{8}c_{i}d_{j}\left|i\rangle ⊗\right|j^{\prime }\rangle ={\left[\begin{array}{cccc} b_{1} & b_{2} & \cdots & b_{8} \end{array}\right]}^{T},$$

where $b_{i}={\left[\begin{array}{cccc} c_{i}d_{1} & c_{i}d_{2} & \cdots & c_{i}d_{8} \end{array}\right]}^{T}=c_{i}\sum_{j=1}^{8}d_{j}\left|j^{\prime }\rangle =c_{i}\right|\psi^{\prime }\rangle$. Then

(8) $$U|\psi \rangle ⊗|\psi^{\prime }\rangle ={\left[\begin{array}{cccc} U_{1}b_{1} & U_{2}b_{2} & \cdots & U_{8}b_{8} \end{array}\right]}^{T}={\left[\begin{array}{cccc} c_{1}U_{1}|\psi^{\prime }\rangle & c_{2}U_{2}|\psi^{\prime }\rangle & \cdots & c_{8}U_{8}|\psi^{\prime }\rangle \end{array}\right]}^{T}.$$

We can see |ψ〉 (corresponding to the original network) works as the controller and the proposed $U_{i}$ mainly operates on $|\psi^{\prime }\rangle$ (corresponding to the mirror network). For example, $c_{i}=0$ simply turns off $U_{i}$ while $c_{i}\neq 0$ turns on and adjusts $U_{i}$.

To see the effects of $U_{i}$ on $|\psi^{\prime }\rangle$, it is more convenient to view $|\psi^{\prime }\rangle$ as a 3-qubit superposition state spanned by $|z^{\prime }y^{\prime }x^{\prime }\rangle$, noting the following fact:

(9) (U⊗V⊗W)(|α〉⊗|β〉⊗|γ〉)=(U|α〉)⊗(V|β〉)⊗(W|γ〉).

Each $U_{i}$ is constructed based on the two-dimensional rotation matrix:

(10) $$U_{\theta }=\left[\begin{array}{cc} \cos\theta & -\sin\theta \\ \sin\theta & \cos\theta , \end{array}\right]$$

where θ is the rotation angle. We will add subscript to θ to indicate which agent is infected. For example, $\theta_{x}$ indicates that the rotation is operated on |x〉 and $\theta_{yx}$ indicates that the rotation of counteraction is operated on |y〉 controlled by |x〉.The compositions of each $U_{i}$ are introduced as follows.

#### 2.2.1. $U_{1}$

$U_{1}$ is controlled by the original |ψ〉 through $c_{1}$, the coefficient of |000〉. $U_{1}$ is constructed as follows:

(11) $$U_{X}=I_{4}⊗U_{\theta_{x}}$$

(12) $$U_{Y}=I_{2}⊗U_{\theta_{y}}⊗I_{2}$$

(13) $$U_{Z}=U_{\theta_{z}}⊗I_{4}$$

(14) $$U_{1}=U_{Z}\cdot U_{Y}\cdot U_{X}.$$

$U_{X}$ rotates the third qubit by angle $\theta_{x}$ and leaves the first two qubits intact, that is, $U_{X}$ only infects $A_{1}$ mirror by a small amount. Similarly, $U_{Y}$ only infects $A_{2}$ mirror by angle $\theta_{y}$ and $U_{Z}$ only infects $A_{3}$ mirror by angle $\theta_{z}$. Therefore, $U_{1}$ infects all agent mirrors incrementally. Therefore, $U_{1}$ infects all agent mirrors incrementally, simultaneously, and independently.

#### 2.2.2. $U_{2}$

$U_{2}$ is controlled by the original |ψ〉 through $c_{2}$, the coefficient of |001〉. $U_{2}$ is constructed as follows:

(15) $$U_{Y}=I_{2}⊗\left(I_{2}⊗M_{0}+U_{\theta_{y}}⊗M_{1}\right)$$

(16) $$U_{X}=I_{2}⊗\left(M_{0}⊗U_{\theta_{x}}+M_{1}⊗I_{2}\right)$$

(17) $$U_{2}=U_{X}\cdot U_{Y}.$$

$U_{Y}$ rotates the second qubit by angle $\theta_{y}$ under the control of the third qubit and leaves the first qubit intact, that is, $U_{Y}$ only infects $A_{2}$ mirror by the full prescribed amount if $A_{1}$ mirror is infected (in state |1〉). Similarly, $U_{X}$ only infects $A_{1}$ mirror by the full prescribed amount if $A_{2}$ mirror is not infected at all. Therefore, $U_{2}$ is the interaction between $A_{1}$ mirror and $A_{2}$ mirror. Note that the order is important: $A_{1}$ mirror firstly controls the infection of $A_{2}$ mirror and then $A_{2}$ mirror controls the infection of $A_{1}$ mirror, which conforms to the setting that the propagation is initiated at $A_{1}$ mirror. First, $A_{2}$ mirror is slightly infected if $A_{1}$ mirror is infected (in state |1〉), which is a natural step. Then, $A_{1}$ mirror is slightly infected if $A_{2}$ mirror is not infected at all. This tricky setting implies that failure to persuade another person would strengthen the idea in your own mind. $U_{2}$ describes the influence exerted by $A_{1}$ mirror on $A_{2}$ mirror and the counteraction received by $A_{1}$ mirror from $A_{2}$ mirror, since only $A_{1}$ is infected in its controller |001〉 and $A_{1}$ can only communicate with $A_{2}$.

#### 2.2.3. $U_{3}$

$U_{3}$ is controlled by the original |ψ〉 through $c_{3}$, the coefficient of |010〉. $U_{3}$ is constructed as follows:

(18) $$U_{Y}=I_{2}⊗\left(M_{0}⊗I_{2}+M_{1}⊗U_{\theta_{x}}\right)$$

(19) $$U_{Z}=\left(I_{2}⊗M_{0}+U_{\theta_{z}}⊗M_{1}\right)⊗I_{2}$$

(20) $$U_{Y_{x}}=I_{2}⊗\left(U_{\theta_{yx}}⊗M_{0}+I_{2}⊗M_{1}\right)$$

(21) $$U_{Y_{z}}=\left(M_{0}⊗U_{\theta_{yz}}+M_{1}⊗I_{2}\right)⊗I_{2}$$

(22) $$U_{3}=U_{Y_{x}}\cdot U_{Y_{z}}\cdot U_{Z}\cdot U_{Y}.$$

Firstly, $U_{Y}$ incrementally infects $A_{1}$ mirror if $A_{2}$ mirror is infected (in state |1〉). Secondly, $U_{Z}$ incrementally infects $A_{3}$ mirror if $A_{2}$ mirror is infected (in state |1〉). Thirdly, $U_{Yz}$ incrementally infects $A_{2}$ mirror if $A_{3}$ mirror is not infected at all. Fourthly, $U_{Yx}$ incrementally infects $A_{2}$ mirror if $A_{1}$ mirror is not infected at all. $U_{3}$ describes the influence exerted by $A_{2}$ mirror on the other two neighbors and the counteraction received by $A_{2}$ mirror from the other two neighbors, since only $A_{2}$ is infected in its controller |010〉.

#### 2.2.4. $U_{4}$

$U_{4}$ is controlled by the original |ψ〉 through $c_{4}$, the coefficient of |011〉. $U_{4}$ is constructed as follows:

(23) $$U_{Y}=\left(M_{0}⊗U_{\theta_{y}}+M_{1}⊗I_{2}\right)⊗I_{2}$$

(24) $$U_{Z}=\left(I_{2}⊗M_{0}+U_{\theta_{z}}⊗M_{1}\right)⊗I_{2}$$

(25) $$U_{4}=U_{Y}\cdot U_{Z}.$$

Firstly, $U_{Z}$ incrementally infects $A_{3}$ mirror if $A_{2}$ mirror is infected (in state |1〉). Then, $U_{Y}$ incrementally infects $A_{2}$ mirror if $A_{3}$ mirror is not infected at all. $U_{4}$ describes the influence exerted by $A_{2}$ mirror on $A_{3}$ mirror and the counteraction received by $A_{2}$ mirror from $A_{3}$ mirror, since only $A_{3}$ is not infected in its controller |011〉 and $A_{3}$ can only communicate with $A_{2}$.

#### 2.2.5. $U_{5}$

$U_{5}$ is controlled by the original |ψ〉 through $c_{5}$, the coefficient of |100〉. $U_{5}$ is constructed as follows:

(26) $$U_{Y}=\left(M_{0}⊗I_{2}+M_{1}⊗U_{\theta_{y}}\right)⊗I_{2}$$

(27) $$U_{Z}=\left(U_{\theta_{z}}⊗M_{0}+I_{2}⊗M_{1}\right)⊗I_{2}$$

(28) $$U_{5}=U_{Z}\cdot U_{Y}.$$

Firstly, $U_{Y}$ incrementally infects $A_{2}$ mirror if $A_{3}$ mirror is infected (in state |1〉). Then, $U_{Z}$ incrementally infects $A_{3}$ mirror if $A_{2}$ mirror is not infected at all. $U_{5}$ describes the influence exerted by $A_{3}$ mirror on $A_{2}$ mirror and the counteraction received by $A_{3}$ mirror from $A_{2}$ mirror, since only $A_{3}$ is infected in its controller |100〉 and $A_{3}$ can only communicate with $A_{2}$.

#### 2.2.6. $U_{6}$

$U_{6}$ is controlled by the original |ψ〉 through $c_{6}$, the coefficient of |101〉. $U_{6}$ is constructed as follows:

(29) $$U_{X}=I_{2}⊗\left(I_{2}⊗M_{0}+U_{\theta_{x}}⊗M_{1}\right)$$

(30) $$U_{Z}=\left(M_{0}⊗I_{2}+M_{1}⊗U_{\theta_{z}}\right)⊗I_{2}$$

(31) $$U_{X_{y}}=I_{2}⊗\left(M_{0}⊗U_{\theta_{xy}}+M_{1}⊗I_{2}\right)$$

(32) $$U_{Z_{y}}=\left(U_{\theta_{zy}}⊗M_{0}+I_{2}⊗M_{1}\right)⊗I_{2}$$

(33) $$U_{6}=U_{Z_{y}}\cdot U_{X_{y}}\cdot U_{Z}\cdot U_{X}.$$

Since only $A_{2}$ is not infected at all in its controller |101〉, $A_{2}$ mirror receives the idea from the two neighbors and then counteracts the two neighbors. Unlike the classical if-control, where the action happens only if the “if” is true, quantum if-control can superpose the action of both “if” and “else”. In other words, quantum if-control allows half-true-half-false.

#### 2.2.7. $U_{7}$

$U_{7}$ is controlled by the original |ψ〉 through $c_{7}$, the coefficient of |110〉. $U_{7}$ is constructed as follows:

(34) $$U_{X}=I_{2}⊗\left(M_{0}⊗I_{2}+M_{1}⊗U_{\theta_{x}}\right)$$

(35) $$U_{Y}=I_{2}⊗\left(U_{\theta_{y}}⊗M_{0}+I_{2}⊗M_{1}\right)$$

(36) $$U_{7}=U_{Y}\cdot U_{X}.$$

Since only $A_{1}$ is not infected at all in its controller |110〉 and $A_{1}$ can only communicate with $A_{2}$, $A_{1}$ mirror receives the idea from $A_{2}$ mirror and then counteracts $A_{2}$ mirror.

#### 2.2.8. $U_{8}$

$U_{8}$ is controlled by the original |ψ〉 through $c_{8}$, the coefficient of |111〉. $U_{8}$ is simply constructed as $U_{8}=I_{8}$ since all agents are infected in the controller |111〉 and the idea propagation has come to an end.

#### 2.2.9. Summary

$U_{1}$ represents the idea initialization, rotating each agent to infected state (|1〉) regardlessly. $U_{2}$ - $U_{7}$ represents the interaction between agents. An infected agent (|1〉) would first try to infect an uninfected agent (|0〉) and then the uninfected agent (|0〉) would try to disinfect the infected agent (|1〉). We will furthermore let the positive rotation angle from |0〉 to |1〉 have larger absolute value than the negative rotation angle from |1〉 to |0〉. With these settings, the infected agent has advantages over the uninfected agent and thus the network will sooner or later be infected.

### 2.3. Initialization, Iteration and Stopping Rule

The initial state of the network is |000〉, that is, all agents being uninfected. The original network works as a controller and the mirrored network is the target to be controlled. We use subscript uppercase *C* to denote the states of the original network and use subscript uppercase *T* to denote the states of the mirrored network. Thus the initial state of the quantum walk is $\psi_{0}=\alpha_{C}⊗\alpha_{T}$ and $\rho_{0}=\psi_{0}\cdot \psi_{0}^{†}$, where the superscript † means taking transpose and complex conjugate and $\alpha_{C}=\alpha_{T}={\left[1\;0\;0\;0\;0\;0\;0\;0\right]}^{T}$, that is, both the original network and the mirrored network start from the all-uninfected state. The outer product $\rho_{0}$ of a superposition state with its Hermitian is called density operator.

We are interested in how long it takes for a new idea to prevail in the network. Thus the stopping state is |111〉, that is, all agents being infected. When this state (denoted by subscript lowercase *s*) is reached, the quantum walk is stopped; Otherwise the quantum walk continues. We use subscript lowercase *c* to represent the states allowing the quantum walk to continue. The measurement operator to get the stopping state is thus

(37) $$M_{s}=\mathrm{diag}\left(\left[\begin{array}{cccccccc} 0 & 0 & 0 & 0 & 0 & 0 & 0 & 1 \end{array}\right]\right).$$

Correspondingly, the measurement operator to get the states to continue, is $M_{c}=I_{8}-M_{s}$.

Suppose we have physical systems *A* and *B*, whose state is described by a density operator $\rho^{AB}$. The reduced density operator for system *A* is defined by

(38) $$\rho^{A}\equiv {\mathrm{Tr}}_{B}\left(\rho^{AB}\right),$$

where ${\mathrm{Tr}}_{B}$ is a map of operators known as the partial trace over system *B*. The partial trace is defined by

(39) $${\mathrm{Tr}}_{B}\left(\right|a_{1}\rangle a_{2}\left|⊗\right|b_{1}\rangle b_{2}\left|)\equiv \right|a_{1}\rangle a_{2}\left|\mathrm{Tr}(\right|b_{1}\rangle b_{2}\left|\right),$$

where $|a_{1}\rangle$ and $|a_{2}\rangle$ are any two vectors in the state space of *A*, and $|b_{1}\rangle$ and $|b_{2}\rangle$ are any two vectors in the state space of *B*. The trace operation appearing on the right hand side is the usual trace operation for system *B*, so $\mathrm{Tr}(|b_{1}\rangle b_{2}\left|\right)=b_{2}|b_{1}\rangle$.

#### 2.3.1. First Step

(40) $$\rho_{T}\left(1\right)={\mathrm{Tr}}_{C}\left(U\cdot \rho_{0}\cdot U^{†}\right)$$

(41) $$p_{c}\left(1\right)=\mathrm{Tr}\left(M_{c}\cdot \rho_{T}\left(1\right)\cdot M_{c}\right)$$

(42) $$\rho_{c}\left(1\right)=\frac{M_{c}\cdot \rho_{T}\left(1\right)\cdot M_{c}}{p_{c}\left(1\right)}$$

(43) $$P_{c}\left(1\right)=p_{c}\left(1\right)$$

(44) $$P_{s}\left(1\right)=1-p_{c}\left(1\right).$$

Equation (40) applies the first transition on the composite state $\rho_{0}$ of the original network (controller) and the mirror network (target), and then reduces the resulting state to the state consisting only of the target state by taking partial trace over the control state. $p_{c}\left(1\right)$ is the probability to continue the quantum walk when the reduced/target/mirror state is measured and the resulting state is just $\rho_{c}\left(1\right)$. Thus $P_{c}\left(1\right)$ and $P_{s}\left(1\right)$ are the probabilities to continue and stop the quantum walk at the first step, respectively.

#### 2.3.2. Iteration

Given a very small ϵ>0, for t>1, while $\sum_{\tau =1}^{t}P_{s}\left(\tau \right)<1-\epsilon$, using lifting operation

(45) $$\rho \left(t-1\right)=\rho_{c}\left(t-1\right)⊗\rho_{c}\left(t-1\right)$$

(46) $$\rho_{T}\left(t\right)={\mathrm{Tr}}_{C}\left(U\cdot \rho \left(t-1\right)\cdot U^{†}\right)$$

(47) $$p_{s}\left(t\right)=\mathrm{Tr}\left(M_{s}\cdot \rho_{T}\left(t\right)\cdot M_{s}\right)$$

(48) $$p_{c}\left(t\right)=\mathrm{Tr}\left(M_{c}\cdot \rho_{T}\left(t\right)\cdot M_{c}\right)$$

(49) $$\rho_{c}\left(t\right)=\frac{M_{c}\cdot \rho_{T}\left(t\right)\cdot M_{c}}{p_{c}\left(t\right)}$$

(50) $$P_{c}\left(t\right)=P_{c}\left(t-1\right)\cdot p_{c}\left(t\right)$$

(51) $$P_{s}\left(t\right)=P_{c}\left(t-1\right)\cdot p_{s}\left(t\right).$$

The state $\rho_{c}\left(t-1\right)$ from the last step is a reduced state. Thus, Equation (45) lifts it by taking tensor product with itself to apply operator *U*. Equation (46) applies *U* to ρ(t−1) and reduces it again to the target state by taking partial trace over the controller state. In each time step, we lift the state to higher dimension, apply *U* to the lifted state, and then reduce the state to the original dimension. Lifting the state and applying the higher dimension *U* consisting of various controlled-gate allow us to model the asymmetric interactions between agents. Then we have to reduce the state to the original dimension since the state of the network is in the original dimension and various probabilities must be calculated in the original dimension. $p_{c}\left(t\right)$ and $p_{s}\left(t\right)$ are the probabilities to continue and stop the quantum walk when the reduced/target/mirror state is measured, respectively. The resulting continuing state is just $\rho_{c}\left(t\right)$. Thus $P_{c}\left(t\right)$ and $P_{s}\left(t\right)$ are the probabilities to continue and stop the quantum walk at step *t*, respectively. The iteration implies that the quantum walk satisfies the Markov property, that is, the state at step t+1 only depends on the state at step *t*.

**Theorem** **1.** *Given any ϵ>0, there exists an integer t such that $\sum_{\tau =1}^{t}P_{s}\left(\tau \right)\geq 1-\epsilon$ if for some δ>0, $p_{c}\left(\tau \right)>1-\delta$ only for finitely many τ. $\sum_{\tau =1}^{t}P_{s}\left(\tau \right)=1$ if $p_{c}\left(t\right)=0$.*

**Proof.**  $$\begin{array}{cc} \; & P_{s}\left(1\right)+P_{s}\left(2\right)+P_{s}\left(3\right)+P_{s}\left(4\right)+\cdots +P_{s}\left(t\right)\\ = & 1-p_{c}\left(1\right)+p_{c}\left(1\right)\left(1-p_{c}\left(2\right)\right)+p_{c}\left(1\right)p_{c}\left(2\right)\left(1-p_{c}\left(3\right)\right)\\ + & p_{c}\left(1\right)p_{c}\left(2\right)p_{c}\left(3\right)\left(1-p_{c}\left(4\right)\right)+\cdots +p_{c}\left(1\right)p_{c}\left(2\right)\cdots p_{c}\left(t-1\right)\left(1-p_{c}\left(t\right)\right)\\ = & 1-p_{c}\left(1\right)+p_{c}\left(1\right)-p_{c}\left(1\right)p_{c}\left(2\right)+p_{c}\left(1\right)p_{c}\left(2\right)-p_{c}\left(1\right)p_{c}\left(2\right)p_{c}\left(3\right)\\ + & p_{c}\left(1\right)p_{c}\left(2\right)p_{c}\left(3\right)-p_{c}\left(1\right)p_{c}\left(2\right)p_{c}\left(3\right)p_{c}\left(4\right)+\cdots -p_{c}\left(1\right)p_{c}\left(2\right)\cdots p_{c}\left(t-1\right)p_{c}\left(t\right)\\ = & 1-p_{c}\left(1\right)p_{c}\left(2\right)\cdots p_{c}\left(t-1\right)p_{c}\left(t\right). \end{array}$$

Since $0\leq p_{c}\left(\tau \right)\leq 1$ for each τ and for some δ>0, $p_{c}\left(\tau \right)>1-\delta$ only for finitely many τ, there exists an integer *t* such that $p_{c}\left(1\right)p_{c}\left(2\right)\cdots p_{c}\left(t-1\right)p_{c}\left(t\right)<\epsilon$.  □

*U* is an incremental infection operator by construction, so the quantum walk will stop, that is, almost all agents will be infected, in finite number of steps. Thus Pr(quantum walk stops in finite steps) = 1. Suppose the quantum walk stops in *N* steps. Even if we do not know *N*, we can keep on summing $P_{s}\left(\tau \right)$ since we know it will sum up to more than 1−ϵ in finite summations.

## 3. Ring Graph of Agent Connections

### 3.1. Three Agents

This is actually a complete graph where each agent can communicate with the other two agents. Such a ring graph has more connections than the previous line graph. The $U_{2}$, $U_{4}$, $U_{5}$, and $U_{7}$ gates are different for these two graphs, while the other $U_{i}$’s are the same.

In the line graph, $A_{1}$ can only infect $A_{2}$. In the ring graph, $A_{1}$ can infect both $A_{2}$ and $A_{3}$. Three options exist: $A_{1}$ can infect $A_{2}$ and $A_{3}$ simultaneously (e.g., by giving a lecture), $A_{1}$ can infect $A_{2}$ first and then infect $A_{3}$, and $A_{1}$ can infect $A_{3}$ first and then infect $A_{2}$. Three options also exist in the reverse way. Typically, a person can only listen to one person speaking. However, two people disinfects one person simultaneously is still possible. For example, two people can jointly write one letter to a person. Here we only consider the most common scene: One person gives a presentation to two people, but the two people give their respective feedback in order.

1. $U_{2}$.
  (52) $$U_{YZ}=I_{4}⊗M_{0}+U_{\theta_{y}}⊗U_{\theta_{z}}⊗M_{1}$$
  (53) $$U_{Xy}=I_{2}⊗\left(M_{0}⊗U_{\theta_{xy}}+M_{1}⊗I_{2}\right)$$
  (54) $$U_{Xz}=M_{0}⊗I_{2}⊗U_{\theta_{xz}}+M_{1}⊗I_{4}$$
  (55) $$U_{2}=U_{Xz}\cdot U_{Xy}\cdot U_{YZ}$$
  Here $A_{2}$ gives feedback first before $A_{3}$ does.
2. $U_{4}$.
  (56) $$U_{Yz}=\left(M_{0}⊗U_{\theta_{y}}+M_{1}⊗I_{2}\right)⊗I_{2}$$
  (57) $$U_{Zy}=\left(I_{2}⊗M_{0}+U_{\theta_{z}}⊗M_{1}\right)⊗I_{2}$$
  (58) $$U_{Xz}=M_{0}⊗I_{2}⊗U_{\theta_{x}}+M_{1}⊗I_{4}$$
  (59) $$U_{Zx}=I_{4}⊗M_{0}+U_{\theta_{z}}⊗I_{2}⊗M_{1}$$
  (60) $$U_{4}=U_{Zx}\cdot U_{Xz}\cdot U_{Yz}\cdot U_{Zy}$$
  Here $A_{2}$ interacts with $A_{3}$ and then $A_{1}$ interacts with $A_{3}$.
3. $U_{5}$.
  (61) $$U_{Y}=\left(M_{0}⊗I_{2}+M_{1}⊗U_{\theta_{y}}\right)⊗I_{2}$$
  (62) $$U_{X}=M_{0}⊗I_{4}+M_{1}⊗I_{2}⊗U_{\theta_{x}}$$
  (63) $$U_{Zy}=\left(U_{\theta_{zy}}⊗M_{0}+I_{2}⊗M_{1}\right)⊗I_{2}$$
  (64) $$U_{Zx}=U_{\theta_{zx}}⊗I_{2}⊗M_{0}+I_{4}⊗M_{1}$$
  (65) $$U_{5}=U_{Zx}\cdot U_{X}\cdot U_{Zy}\cdot U_{Y}$$
  Here $A_{2}$ interacts with $A_{3}$ and then $A_{1}$ interacts with $A_{3}$.
4. $U_{7}$.
  (66) $$U_{Xy}=I_{2}⊗\left(M_{0}⊗I_{2}+M_{1}⊗U_{\theta_{xy}}\right)$$
  (67) $$U_{Xz}=M_{0}⊗I_{4}+M_{1}⊗I_{2}⊗U_{\theta_{xz}}$$
  (68) $$U_{Y}=I_{2}⊗\left(U_{\theta_{y}}⊗M_{0}+I_{2}⊗M_{1}\right)$$
  (69) $$U_{Z}=U_{\theta_{z}}⊗I_{2}⊗M_{0}+I_{4}⊗M_{1}$$
  (70) $$U_{7}=U_{Z}\cdot U_{Xz}\cdot U_{Y}\cdot U_{Xy}.$$
  Here $A_{2}$ interacts with $A_{3}$ and then $A_{1}$ interacts with $A_{3}$.

### 3.2. More Than Three Agents

With *n* agents, there are $2^{n}$ basis states for the network and $2^{n}\times 2^{n}$ basis states for the lifted network. The unitary operator driving the quantum walk is still in the block diagonal form:

(71) $$U=\mathrm{diag}(\left[\begin{array}{cccc} U_{1} & U_{2} & \cdots & U_{2^{n}}, \end{array}\right])$$

where each $U_{i}$ is an $2^{n}\times 2^{n}$ matrix. In the ring graph, $A_{k}$ can only communicate with $A_{k-1}$ and $A_{k+1}$ for k>1. $A_{1}$ can only communicate with $A_{2}$ and $A_{n}$. $A_{n}$ can only communicate with $A_{1}$ and $A_{n-1}$.

The basis state for the network is $|b_{n}\cdots b_{2}b_{1}\rangle$, where each $b_{i}\in \left\{0,1\right\}$ is the local state of $A_{i}$. $U_{i}$ is determined by the corresponding $|b_{n}\cdots b_{2}b_{1}\rangle$. We assume interaction only exists between two connecting agents with different ideas. We further assume that the infected agent firstly tries to infect the non-infected neighbors and then the neighbors counteract. Take the five-agent basis state |01101〉, for example. Interactions exist between $A_{5}$ and $A_{4}$, $A_{3}$ and $A_{2}$, $A_{2}$ and $A_{1}$. The basis states with the least interaction are the all-zero state and the all-one state. The basis states with the most interaction are |0101⋯〉 and |1010⋯〉.

Let $\theta_{ij}$ and $\phi_{ij}$ be the rotation angles of the operators for agent $A_{i}$ being infected by $A_{j}$ and for $A_{i}$ being counteracted (typically disinfected) by $A_{j}$, respectively. Then $\theta_{1}$ represents amount of infection and $\theta_{2}$ represents amount of counteraction (typically disinfection). Algorithm 1 shows how to generate $U_{i}$. In Algorithm 1, if both $A_{k-1}$ and $A_{k+1}$ are lectured by $A_{k}$, $A_{k-1}$ gives feedback first and then $A_{k+1}$ gives feedback for 1<k<n. However, this order can be readily altered. In Algorithm 1, interactions between agents with lower index take place before those between agents with higher index. Again this order can be readily altered. Note that Algorithm 1 is not for the all-zero state and the all-one state. The $U_{1}$ for the all-zero state is

(72) $$U_{1}=\overset{n}{\underset{k=1}{⨂}}U_{\theta_{k}},$$

where $U_{\theta_{k}}$ is the initial infection amount for $A_{k}$.
**Algorithm 1:** Generating $U_{i}$ for *n*-agent ring network (n>3)
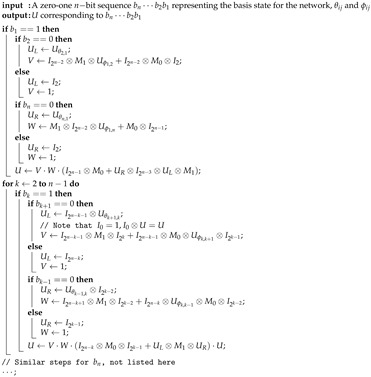


## 4. Results and Discussions

Section 4.1, Section 4.2 and Section 4.3 consider the network in line graph connection. Section 4.4, Section 4.5 and Section 4.6 considers the network in ring graph connection.

### 4.1. No Disinfection

Let $\theta_{x}=0.05$ and $\theta_{y}=\theta_{z}=0.01$ for $U_{1}$. Then $A_{1}$ is more easily infected than $A_{2}$ and $A_{3}$ since $A_{1}$ is the starting point of the propagation. Let $\theta_{x}=0.01$ and $\theta_{y}=0.05$ for $U_{2}$. Then, $\theta_{x}=\theta_{z}=0.05$ and $\theta_{yx}=\theta_{yz}=0.01$ for $U_{3}$, $\theta_{z}=0.05$ and $\theta_{y}=0.01$ for $U_{4}$, $\theta_{z}=0.01$ and $\theta_{y}=0.05$ for $U_{5}$, $\theta_{x}=\theta_{z}=0.025$ and $\theta_{xy}=\theta_{zy}=0.01$ for $U_{6}$, $\theta_{x}=0.05$ and $\theta_{y}=0.01$ for $U_{7}$. With these settings, the first one to talk, whether she tries to infect or disinfect, has more influence than the second one to talk. Thus the influence of the sender has on the receiver is always more powerful than the counteraction of the receiver has on the sender. Note that the sender may send infection or disinfection.

**Figure 1:**
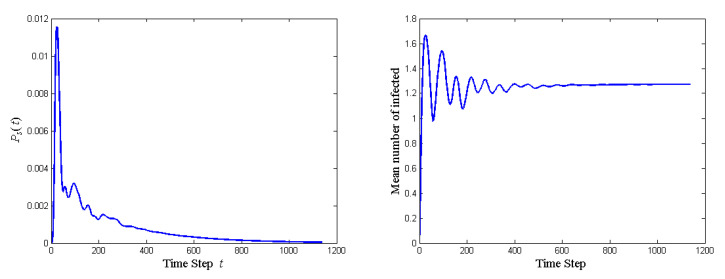
The probability of stopping the quantum walk and the mean number of agents affected at each step until the quantum walk stops. $P_{s}\left(t\right)$ can be viewed as a probability density function whose support is a subset of the time domain. $P_{s}\left(t\right)$ is the probability that the quantum walk stops at time *t*. Summing $P_{s}\left(\tau \right)$ up to time *t* is the probability mass function that the quantum walk stops before time *t*. Note that $P_{s}\left(1140\right)=0$ does not mean that the quantum walk stops at t=1140. Instead, it means that the quantum walk must not stop at t=1140 (must stop before t=1140 actually).

We take ϵ=0.01 in the stopping rule. The probability of stopping the quantum walk, that is, to reach the all-infected state, $P_{s}\left(t\right)$, and the mean number of agents affected, at each time step until the quantum walk stops is shown in Figure 1. The time evolution curves have many sharp oscillations, indicating many back-and-forth arguments in the network. It takes 1139 steps to stop the quantum walk. $P_{s}\left(t\right)$ is very low, smaller than 0.012, in each step. $P_{s}\left(t\right)$ rises rapidly to the peak value at the 24th step, and then drops rapidly. After 400 steps, $P_{s}\left(t\right)$ just decreases slowly. The propagation of idea is fast only at the very beginning and is slow afterwards. Note that $\rho_{c}\left(t\right)$ represents the state of the social network and its diagonal elements are the probabilities of getting the basis state. Thus the (1,1) entry of $\rho_{c}\left(t\right)$ is the probability that the network is in non-infected state (|000〉) at step *t*, (2,2) entry is the probability of only $A_{1}$ is infected (|001〉), and so forth. The mean number of agents affected at step *t* is $\left[0\;1\;1\;2\;1\;2\;2\;3\right]\mathrm{diag}(\rho_{c}\left(t\right))$ where $\mathrm{diag}(\rho_{c}\left(t\right))$ is the column vector formed by diagonal entries of $\rho_{c}\left(t\right)$. The mean number oscillates much in the first 200 steps and goes to a steady value of 1.27 after 600 steps. Table 1 lists the probabilities of each state at the last step. The distribution is quite uniform. Note that the probability of all infected (000) is 0 since $\rho_{c}\left(t\right)$ is obtained by projection removing 000 in Equation (49).

### 4.2. Stubborn Conservative Guy in the Middle

Consider the following case: $A_{2}$ is conservative and stubborn while $A_{1}$ and $A_{3}$ are open-minded and compliant. Thus it is easier for $A_{2}$ to persuade $A_{1}$ than it is for $A_{1}$ to persuade $A_{2}$.

Set $\theta_{x}=\theta_{z}=0.05$ and $\theta_{y}=0.01$ for $U_{1}$.

Set $\theta_{y}=0.01$ and $\theta_{x}=-0.02$ for $U_{2}$. Thus it is easier for $A_{2}$ to disinfect $A_{1}$ than it is for $A_{1}$ to infect $A_{2}$.

Set $\theta_{x}=\theta_{z}=0.02$ and $\theta_{yx}=\theta_{yz}=-0.01$ for $U_{3}$. If $A_{2}$ is infected, he can infect his two neighbors powerfully. If $A_{1}$ and $A_{3}$ are not infected at all, they can slightly disinfect $A_{2}$.

Set $\theta_{z}=0.02$ and $\theta_{y}=-0.01$ for $U_{4}$. If $A_{2}$ is infected, he can infect $A_{3}$ powerfully. If $A_{3}$ is not infected at all, she can slightly disinfect $A_{2}$.

Set $\theta_{y}=0.01$ and $\theta_{z}=-0.02$ for $U_{5}$. If $A_{3}$ is infected, she can infect $A_{2}$ slightly. If $A_{2}$ is not infected at all, he can disinfect $A_{3}$ powerfully.

Set $\theta_{x}=\theta_{z}=0.01$ and $\theta_{xy}=\theta_{zy}=-0.02$ for $U_{6}$. If $A_{2}$ is not infected at all, he can disinfect his two neighbors powerfully. If $A_{1}$ and $A_{3}$ are infected, they can slightly infect $A_{2}$.

Set $\theta_{y}=-0.01$ and $\theta_{x}=0.02$ for $U_{7}$. Thus it is easier for $A_{2}$ to infect $A_{1}$ than it is for $A_{1}$ to disinfect $A_{2}$.

**Figure 2:**
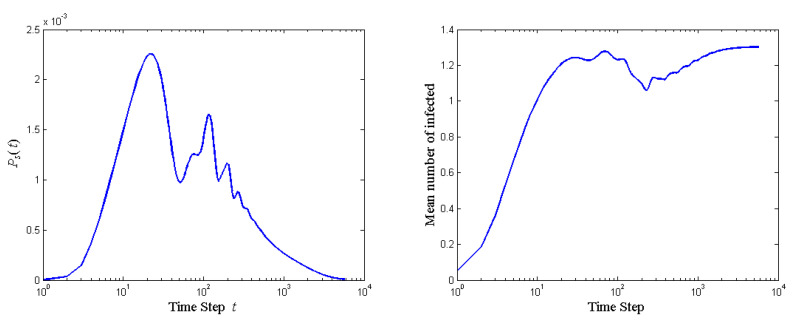
The probability of stopping the quantum walk and the mean number of agents affected at each step of the stubborn-middle network.

The probability of stopping the quantum walk and the mean number of agents affected at each step of the stubborn-middle network is shown in Figure 2. Note that the horizontal axis is in log scale since it takes much longer to stop the quantum walk than in the last subsection (5762 vs. 1139). $P_{s}\left(t\right)$ with a stubborn guy in the middle (Figure 2) is much lower than that without a stubborn guy in the middle (Figure 1), since the infection rate is much slower (smaller rotation angle). The mean number of infected in Figure 2 is also smaller than that in Figure 1 in initial stages, but it catches up in later stages. This indicates that the stubborn guy in the middle acts as a barrier to idea propagation only at the initial stages but accelerate the propagation after he is persuaded, since the middle guy is hard to be persuaded by neighbors (small controlled rotation by neighbors) and is influential over neighbors (large controlled rotation by the middle guy).

The results demonstrate that the proposed model is able to model asymmetric interactions between agents with a stubborn guy in the middle. Next we will further demonstrate this by applying the proposed quantum walk to asymmetric interactions between agents with a compliant guy in the middle.

### 4.3. Compliant Open-Minded Guy in the Middle

Consider the following case: $A_{2}$ in this subsection is more compliant and open-minded than $A_{2}$ in the last subsection.

Set $\theta_{x}=\theta_{y}=\theta_{z}=0.05$ for $U_{1}$. Here the three agents are equally open-minded.

Set $\theta_{y}=0.02$ and $\theta_{x}=-0.01$ for $U_{2}$. Thus it is easier for $A_{1}$ to infect $A_{2}$ than it is for $A_{2}$ to disinfect $A_{1}$.

Set $\theta_{x}=\theta_{z}=0.02$ and $\theta_{yx}=\theta_{yz}=-0.01$ for $U_{3}$. If $A_{2}$ is infected, he can infect his two neighbors powerfully. If $A_{1}$ and $A_{3}$ are not infected at all, they can slightly disinfect $A_{2}$.

Set $\theta_{z}=0.02$ and $\theta_{y}=-0.01$ for $U_{4}$. If $A_{2}$ is infected, he can infect $A_{3}$ powerfully. If $A_{3}$ is not infected at all, she can slightly disinfect $A_{2}$.

Set $\theta_{y}=0.01$ and $\theta_{z}=-0.02$ for $U_{5}$. If $A_{3}$ is infected, she can infect $A_{2}$ slightly. If $A_{2}$ is not infected at all, he can disinfect $A_{3}$ powerfully.

Set $\theta_{x}=\theta_{z}=0.02$ and $\theta_{xy}=\theta_{zy}=-0.01$ for $U_{6}$. If $A_{2}$ is not infected at all, he can slightly disinfect his two neighbors. If $A_{1}$ and $A_{3}$ are infected, they can infect $A_{2}$ powerfully.

Set $\theta_{y}=-0.01$ and $\theta_{x}=0.02$ for $U_{7}$. Thus it is easier for $A_{2}$ to infect $A_{1}$ than it is for $A_{1}$ to disinfect $A_{2}$.

**Figure 3:**
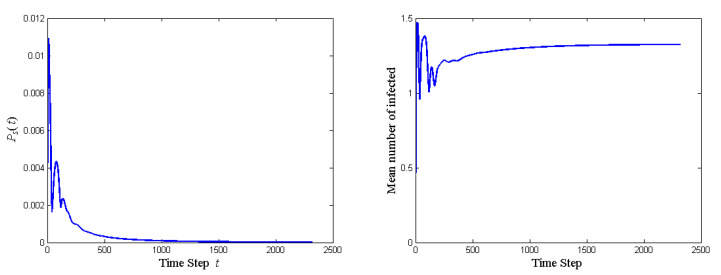
The probability of stopping the quantum walk and the mean number of agents affected at each step of the compliant-middle network.

The probability of stopping the quantum walk and the mean number of agents affected at each step of the compliant-middle network is shown in Figure 3. $P_{s}\left(t\right)$ with a compliant guy in the middle (Figure 3) is slightly lower than that in the no-disinfection case (Figure 1), but it takes much longer to stop the quantum walk, since the infection rate is much lower. The lower infection rate is not exactly due to smaller rotation angles. Instead, it is due to disinfection (negative rotation angle) compared to the no-disinfection case. The mean number of infected in Figure 3 is less oscillatory and smaller than that in Figure 1 in initial stages, but it catches up in later stages. This indicates that the compliant guy in the middle facilitates idea propagation throughout the quantum walk. The smooth propagation brought by the middle guy can be explained as follows. Although the compliant guy is not very influential over neighbors (large controlled rotation by the middle guy) and thus does not accelerate the propagation after he is persuaded, but the middle guy is easily persuaded by neighbors (small controlled rotation by neighbors).

The results in the previous three subsections demonstrate that the proposed model is able to model asymmetric interactions between agents with various personalities communicating in a line graph structure. Next we will further demonstrate this by applying the proposed quantum walk to asymmetric interactions between agents communicating in a ring graph structure

### 4.4. Ring Graph

**Figure 4:**
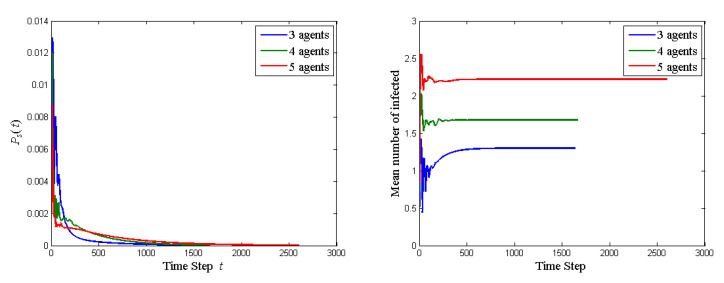
The probability of stopping the quantum walk and the mean number of agents affected at each step of the quantum walk on the ring graph.

Set all $\theta_{ij}=0.05$ and all $\phi_{ij}=-0.01$. The probability of stopping the quantum walk and the mean number of agents affected at each step of the quantum walk on the ring graph are shown in Figure 4. As the size of the network increases, $P_{s}\left(t\right)$ drops more rapidly in the first few steps but drops more slowly in later steps, and it takes longer for the new idea to prevail the whole network. This can be explained by the fact that each agent is initially exposed to the new idea (see Equation (72)) and thus larger networks have more agents initializing the propagation, but it still takes longer time for more agents to end at the same basis state. The mean numbers of infected evolving with time with different network size has the same pattern: oscillating in the first few steps and then converge in later steps. The oscillation in the larger network is less intensive since larger population provides larger buffer and thus there is less back-and-forth changes of minds. With one agent added, the increase in the mean number of infected from 3 to 4 is larger than that from 4 to 5. This can also be explained by the fact that adding an agent is adding an idea initiator and messenger.

Up to this point, we apply the proposed quantum walk model to idea propagation in a social network of agents with various personalities. In such a setting, all agents have the same initial state (not knowing the new idea) and the same final state (accepting the new idea). Next we apply the proposed quantum walk model to group decision making in the next subsection.

### 4.5. Reaching Agreement

In group decision making, the group members have different ideas initially. After the group discussion, an agreement is reached. Consider a binary group decision making problem where each group member’s local state is either 1 or 0, for example, agreeing on passing the proposal or disagreeing. Suppose the decision can be made only if the group has reached an agreement, that is, all members are 1 or all members are 0. The special case of all members having the same initial state is trivial since no discussion is needed and the decision can be made immediately. The goal of this subsection is to investigate the influence the initial distribution of tendencies of the group members on the final decision.

The initial state of the network is no longer |00⋯0〉. Actually the initial state is the influencing factor that we want to investigate. Take a 3-member group as example. If $A_{1}$ and $A_{2}$ is completely sure of option 1 while $A_{3}$ is completely sure of option 0, then the initial state is |011〉. This is a special case of all agents being completely sure of one option. Actually an agent may not be completely sure. In this case, the initial local state of the agent is a superposition state over |0〉 and |1〉. Then the initial state of the *n*-agent network is the tensor product of the initial local states of the agents:

(73) $$\alpha_{C}=\alpha_{T}=\overset{n}{\underset{k=1}{⨂}}\alpha_{k}\left(0\right),$$

where

(74) $$\alpha_{k}\left(t\right)=c_{k}\left(t\right)\left|0\rangle +d_{k}\left(t\right)\right|1\rangle$$

is the local state of $A_{k}$. Again we lift the network by tensor product with its mirror so that one works as controller and the other works as target. The initial control state and the initial target state are the same. The initial state of the quantum walk is still given by $\psi_{0}=\alpha_{C}⊗\alpha_{T}$ and $\rho_{0}=\psi_{0}\cdot \psi_{0}^{†}$.

We are also interested in how long it takes for a group to reach agreement. Note that now we have two stopping states, namely, |00⋯0〉 and |11⋯1〉. All the group members take the same option. When either state is reached, the quantum walk is stopped; Otherwise the quantum walk continues. The measurement operator to get the stopping state is thus

(75) $$M_{s}=\left(\left[\begin{array}{ccccc} 1 & 0 & \cdots & 0 & 1 \end{array}\right]\right)$$

Correspondingly, the measurement operator to get the states to continue, is $M_{c}=I_{2^{n}}-M_{s}$ for an *n*-member group.

The first step of the quantum walk is still given by Equations (40)–(44) and the iteration is still given by Equations (45)–(51). These equations are valid for *n*-agent networks.

In the new idea propagation setting, $U_{1}$ controlled by |00⋯0〉 just infects each agent by a small amount. Let *n* be the number of agents In the group decision making setting, we set $U_{1}=I_{2^{n}}$ since the group members have initial opinions related to the decision and there is no need to initialize.

In group decision making, besides the time to stop the quantum walk (resp. to make the decision), we are also interested in the final state (resp. which decision is to be made). Suppose the quantum walk stops at step $t_{f}$. Let $\rho_{T}\left(t\right)$ and $p_{s}\left(t\right)$ be as given by Equations (46) and (47), respectively. Then the final state is

(76) $$\rho_{f}=\frac{M_{s}\cdot \rho_{T}\left(t_{f}\right)\cdot M_{s}}{p_{s}\left(t_{f}\right)}$$

The probability to reach an agreement of taking option *i* (i=0,1) is

(77) $$P_{i}=\mathrm{Tr}\left(M_{i}\cdot \rho_{f}\cdot M_{i}\right),$$

where $M_{0}$ is $2^{n}\times 2^{n}$ matrix with all entries zero except (1, 1) entry being 1 and $M_{1}$ is $2^{n}\times 2^{n}$ matrix with all entries zero except ($2^{n}$, $2^{n}$) entry being 1.

**Figure 5:**
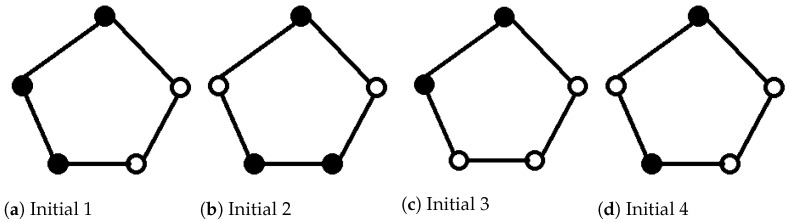
The initial state configurations in the 5-member group (Solid: option 1; Hollow: option 0).

Consider a 5-member group with connections in a ring shape. The initial state configurations in the group are shown in Figure 5 where the solid circle represents a member initially completely sure of taking option 1 and the hollow circle represents a member initially completely sure of taking option 0. Besides these four initial configurations, we consider one more configuration, called Initial 5. In Figure 5a, let the solid circle and the hollow circle represent the state $\sqrt{0.2}\left|0\rangle +\sqrt{0.8}\right|1\rangle$ and the state $\sqrt{0.9}\left|0\rangle +\sqrt{0.1}\right|1\rangle$, respectively. Thus three members are 80% sure of taking option 1 while two agents are 10% sure of taking option 1.

**Figure 6:**
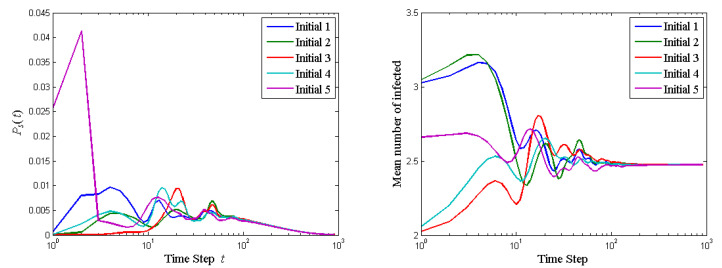
The probability of stopping the quantum walk and the mean number of agents affected at each step of the quantum walk on the 5-member ring graph with various initial state configurations.

Set al.l $\theta_{ij}=0.05$ and all $\phi_{ij}=-0.05$. Thus, all members are equally powerful in persuading the neighbors and equally likely to be persuaded by neighbors. The probability of stopping the quantum walk and the mean number of agents affected at each step of the quantum walk on the 5-member ring graph with various initial state configurations are shown in Figure 6. The quantum walk has certain ergodicity properties: $P_{s}\left(t\right)$ and mean number of infected member (taking option 1) are different in the first tens of steps but they coincide after 100 steps. The final state is superpositioned over |00000〉 and |11111〉. The probability to take option 1 (measurement result being |11111〉) is 0.7488, the same for all these initial configurations. This can be explained by the mechanism of Algorithm 1: In each time step, the interaction occurs in order clockwise (See Figure 5) between two agents. The interactions do not occur simultaneously. What’s more, the interaction always starts from the top solid node in Figure 5 influencing the hollow node on the right side. The agent who first talks has advantage over those who talk later. Therefore, the advantage of talking first and the discussion procedure dominate the effect of initial opinions.

### 4.6. Interference Effects

One important reason for exploring quantum networks is that they can produce interference effects when comparing a system that is disturbed by measurements to a system that is not. To see the interference effect in the quantum walk on social network, again take the 5-member group decision making ring network as example. Note that although Equation (42) and Equation (49) perform measurements, they do not produce interference effects on the intermediate states since they do not affect a particular intermediate state. We can let the quantum walk evolve until the stopping criterion is reached. We can also measure the quantum walk in the half-way before the stopping criterion is reached and then let the quantum walk evolve again for the next half. Such an intermediate measurement is of practical significance. In the middle of the group discussion, each member may be asked to vote. This voting is not used for group decision making, but rather used as an indication of agreement. Note that we can measure at any intermediate step to see the interference effect. Measure at exactly half-way is just one of the many options.

**Figure 7:**
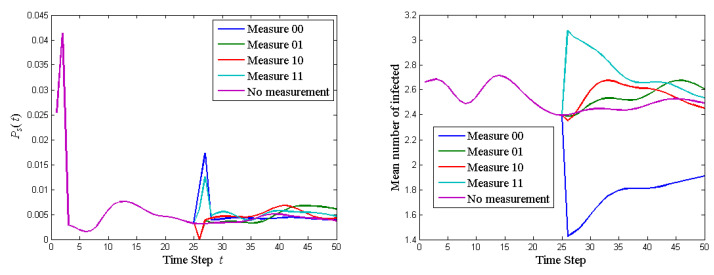
The probability of stopping the quantum walk and the mean number of agents affected at each step of the quantum walk on the 5-member ring graph with various results of half-way measurement on $A_{1}$ and $A_{2}$.

Consider the configuration of Initial 5 in the last subsection. Without half-way measurement, we let the quantum walk evolve for 50 steps. To see the interference effect, we measure the quantum walk half-way. At the 25th step, we only measure $A_{1}$ and $A_{2}$. Then the local states of $A_{1}$ and $A_{2}$ collapse to basis states (|1〉=|00〉, |2〉=|01〉, |3〉=|10〉, |4〉=|11〉) while the other agents’ states are still superpositioned. We can measure them with four measurement operators: $I_{8}⊗M_{i}$, where $M_{i}$ is a 4×4 matrix with all entries zero except the *i*th diagonal entry being 1 for i=1,2,3,4. The probability to get |i〉 is $\mathrm{Tr}(\left(I_{8}⊗M_{i}\right)\cdot \rho_{T}\left(25\right)\cdot \left(I_{8}⊗M_{i}\right))$. Then we let the quantum walk evolve for the remaining 25 steps. The probability of stopping the quantum walk and the mean number of agents affected at each step of the quantum walk on the 5-member ring graph with various results of half-way measurement on $A_{1}$ and $A_{2}$ are shown in Figure 7. With half-way measurement, the measured agents are forced to make a decision and a new path of quantum walk is produced. Four possible new paths of quantum walk can be produced for four possible results of measurement. The partially collapsed new paths deviate from the original fully superpositioned path. Due to the ergodicity of the proposed quantum walk, all paths will eventually converge to the same path. If the group discussion can go on without time constraint, then interference effects would not affect the final decision. However, with constraint on discussion time, interference effect cannot be ignored. At the 25th step, the probabilities of $A_{1}$ and $A_{2}$ choosing 00, 01, 10, 11 are the 1st, 2nd, 3rd, 4th entry of u=[0.1870,0.2479,0.2719,0.2932], respectively. At the 50th step, the probability of all members choosing option 1 for the four new quantum walks are 1st, 2nd, 3rd, 4th entry of v=[0.2378,0.8038,0.6974,0.8033], respectively. According to classical probability, the probability for the group to choose option 1 at the 50th step is u·v=0.6689. However, the probability for the group to choose option 1 without half-way measurement at the 50th step is 0.7602. This violation of the classical Bayes rule is the so-called quantum interference effect. The reason for the interference effect is that the quantum state evolution is based on amplitude instead of amplitude magnitude squared (probability).

## 5. Conclusions

In this paper, we have shown how a quantum walk model can be used to describe the spreading of ideas in a network and the formation of agreement in group decision making. In particular, we have considered networks where connections between agents are in a line graph and in a ring graph. The analysis was based on the use of controlled unitary operators in quantum computing where the original network is used as controller and the mirror network is used as target. We have also discussed the interference effects when two agents are forced to make decisions in the half-way and how this modifies the probability of choosing a particular final option.

Our analysis opens the way to many possible applications, from the application of different stopping rules, to the possibility of modeling special classes of agents (e.g., the so-called influencers) in the network. These are only a few of the possible extensions of the ideas discussed here.

## Figures and Tables

**Table 1:** Probabilities of each state at the last step.

State	000	001	010	011	100	101	110	111
Pr	0.1525	0.1442	0.1295	0.1379	0.1484	0.1439	0.1436	0

## Data Availability

Not Applicable.
